# Comparison of Prediction Models Based on Machine Learning for the Compressive Strength Estimation of Recycled Aggregate Concrete

**DOI:** 10.3390/ma15103430

**Published:** 2022-05-10

**Authors:** Kaffayatullah Khan, Waqas Ahmad, Muhammad Nasir Amin, Fahid Aslam, Ayaz Ahmad, Majdi Adel Al-Faiad

**Affiliations:** 1 Department of Civil and Environmental Engineering, College of Engineering, King Faisal University, Al-Ahsa 31982, Saudi Arabia; mgadir@kfu.edu.sa; 2 Department of Civil Engineering, COMSATS University Islamabad, Abbottabad 22060, Pakistan; waqasahmad@cuiatd.edu.pk; 3 Department of Civil Engineering, College of Engineering in Al-Kharj, Prince Sattam bin Abdulaziz University, Al-Kharj 11942, Saudi Arabia; f.aslam@psau.edu.sa; 4 MaREI Centre, Ryan Institute and School of Engineering, College of Science and Engineering, National University of Ireland Galway, H91 HX31 Galway, Ireland; a.ahmad8@nuigalway.ie; 5 Department of Chemical Engineering, College of Engineering, King Faisal University, Al-Ahsa 31982, Saudi Arabia; malfaiad@kfu.edu.sa

**Keywords:** recycled concrete aggregate, compressive strength, green concrete, machine learning, decision tree, gradient boosting, bagging regressor

## Abstract

Numerous tests are used to determine the performance of concrete, but compressive strength (CS) is usually regarded as the most important. The recycled aggregate concrete (RAC) exhibits lower CS compared to natural aggregate concrete. Several variables, such as the water-cement ratio, the strength of the parent concrete, recycled aggregate replacement ratio, density, and water absorption of recycled aggregate, all impact the RAC’s CS. Many studies have been carried out to ascertain the influence of each of these elements separately. However, it is difficult to investigate their combined effect on the CS of RAC experimentally. Experimental investigations entail casting, curing, and testing samples, which require considerable work, expense, and time. It is vital to adopt novel methods to the stated aim in order to conduct research quickly and efficiently. The CS of RAC was predicted in this research utilizing machine learning techniques like decision tree, gradient boosting, and bagging regressor. The data set included eight input variables, and their effect on the CS of RAC was evaluated. Coefficient correlation (R^2^), the variance between predicted and experimental outcomes, statistical checks, and k-fold evaluations, were carried out to validate and compare the models. With an R^2^ of 0.92, the bagging regressor technique surpassed the decision tree and gradient boosting in predicting the strength of RAC. The statistical assessments also validated the superior accuracy of the bagging regressor model, yielding lower error values like mean absolute error (MAE) and root mean square error (RMSE). MAE and RMSE values for the bagging model were 4.258 and 5.693, respectively, which were lower than the other techniques employed, i.e., gradient boosting (MAE = 4.956 and RMSE = 7.046) and decision tree (MAE = 6.389 and RMSE = 8.952). Hence, the bagging regressor is the best suitable technique to predict the CS of RAC.

## 1. Introduction

Worldwide, concrete is the most utilized material in the building sector [1,2,3,4,5,6]. Its appeal originates from several characteristics, including its minimal expense, water and heat resistance, and flexibility to a variety of shapes and sizes [7,8,9,10,11,12,13]. Concrete might be used to build almost every sort of structure [14,15]. Concrete is composed of three fundamental components: aggregates, cement, and water [16,17,18]. Amongst these ingredients, aggregate is significant as it makes up around 60–75% of the overall volume of concrete [19]. Moreover, the fast growth of industrialization and urbanization has made concrete the least eco-friendly material because it uses the most natural resources. Concrete is crucial to a country’s economic prosperity due to its widespread use. It utilizes around 20,000 million tons of raw materials (natural aggregates) every year [20]. Moreover, the mining and processing natural aggregates (NAs) requires considerable energy and results in increased CO_2_ emissions [21]. Thus, increased use of concrete results in rapid depletion of natural resources and increased contamination of the environment [22,23,24]. Now, scholars are focusing their research on the application of alternate materials to natural ones, thereby promoting naturally responsible construction.

Modern infrastructure development necessitates extensive refurbishment of present structures, causing immense volumes of construction and demolition waste (CDW). Due to the crucial nature of CDW, it must be disposed of securely. Two concerns confront the current building sector: dwindling natural resources and a rise in CDW. Both challenges might be addressed concurrently by CDW recycling in the new building. Recycling leftover concrete from CDW has developed into a feasible alternative to NA in concrete [25,26]. Waste concrete is generated in a number of ways, including destroyed structures, abandoned precast concrete members, residual concrete in batching facilities, and concrete samples tested in laboratories [27,28,29,30]. Thus, incorporating recycled concrete aggregates (RCAs) in the building sector will be an economical and eco-friendly way to decrease CDW volume [31,32]. RAs are divided into three categories: recycled brick aggregate, RCA, and recycled mixed (bricks and concrete) aggregate. Meanwhile, RAs include a range of pollutants, including woodblocks, glass, paper fragments, and plastics [33,34]. Presently, RCA is the most often utilized in construction [35,36,37,38,39,40]. Thus, substituting RCAs from CDW for NAs in concrete will encourage sustainable development.

The process of building predictive models for concrete strength is ongoing in order to minimize needless test repetitions and material waste. There are various popular models for simulating the characteristics of concrete, including best-fit curves (based on regression analysis). However, because concrete has a nonlinear behavior [41,42], regression models developed using this approach may not adequately capture the material’s underlying nature. Additionally, regression techniques may underestimate the influence of concrete constituents [43]. Artificial intelligence techniques, such as machine learning (ML), are some of the most advanced modeling approaches employed in the field of civil engineering. These methods model responses using input variables, and the output models are confirmed by experimentation. ML methods are employed to forecast concrete strength [44,45,46,47,48], the performance of bituminous mixtures [49], and the durability of concrete [50,51,52]. The majority of previous ML-based studies have focused on CS prediction for conventional concretes [53,54,55,56,57,58,59], using their physiochemical attributed (e.g., cement content; water content; and mass/volume of admixture and/or mineral additive); only a few articles have focused on the prediction of the characteristics of RAC. Duan et al. [60] used a nonlinear, regression-based ML model, namely an artificial neural network (ANN), to forecast the CS of RAC. Gholampour et al. [61] investigated the applicability of several regression-based ML models for predicting the mechanical properties of RAC. Deshpande et al. [62] employed ANN to predict the CS of RAC, which might possibly be used to estimate MOE when paired with semi-empirical formulae. Behnood et al. [63] predicted the properties of RAC using the M5P model tree technique—a very recent decision tree ML model [64]. Deng et al. [65] predicted the CS of RAC using a convolutional ANN-based deep learning algorithm. Nonetheless, it is critical to note that the most frequently used ML model in prior research is ANN frequently fails to accurately predict outcomes [66,67]. This is because ANN models are based on local optimization and search algorithms (e.g., the back-propagation mechanism used in several neural network-based ML models for parameter optimization) that are highly susceptible to becoming confined in (or around) local minima rather than converging to the global minimum [66]. As a result of this difficulty, when ANN models are retrained, they frequently provide inconsistently or even poorer predictions for the same set of inputs (e.g., using a larger or a different database) [68]. Recent studies have demonstrated that the bagging regressor (BR) and gradient boosting (GB) models based on a modification of the bootstrap aggregation decision tree (DT) algorithm outperforms other standalone ML models in terms of prediction accuracy of concrete CS [69,70,71,72]. These studies credit the BR and GB model’s better prediction performance to its unmatched ability to handle discrete and continuous variables across monotonic and non-monotonic data domains, while simultaneously lowering variance across different subsets of the training data set. Despite the BR and GB model’s benefits, an exhaustive literature analysis revealed that these models have rarely been used to forecast the CS of RAC.

The aim of this work is to determine how ML strategies might be used to anticipate the CS of RAC. One single ML algorithm, DT, and two ensemble ML approaches, GB and BR, were employed. To evaluate the performance of each method, correlation coefficients (R^2^) and statistical tests were carried out. Furthermore, each technique’s validity was confirmed using k-fold evaluation and error dispersals. This research is noteworthy because it predicts the CS of RAC utilizing both single and ensemble ML methods. The experimental explorations require substantial human effort, experimentation expenses, and time for collection, casting, curing, and testing materials. Since a variety of parameters, including waster–cement ratio (w/c), parent concrete strength, recycled aggregate replacement ratio, water absorption, and density, all influence the CS of RAC, and their combined effect is difficult to analyze experimentally. ML techniques are capable of identifying the cumulative influence of their components with minimal effort. ML methods require a data set, which may be gathered from previous research since several investigations have been conducted to determine the CS of RAC. The data collected can then be employed to train ML methods and anticipate material strength. Some previous studies also employed ML methods to estimate the properties of RAC, but with a limited number of data samples and input parameters. For example, Salimbahrami and Shakeri [73] predicted the CS of RAC using the ANN technique with 7 input variables and 124 data samples. Similarly, Duan et al. [74] predicted the CS of RAC with 6 input variables and 209 data points. This study employed different ML techniques from the previous studies and estimated the CS of RAC with 8 input parameters and 638 data points. It is expected that using a higher number of input variables and data points will result in the superior precision of ML techniques. The goal of this research is to determine the most appropriate ML approach for estimating the CS of RAC and the influence of various factors on RAC strength.

## 2. Methods

### 2.1. Data Employed for Modeling

**Figure 1:**
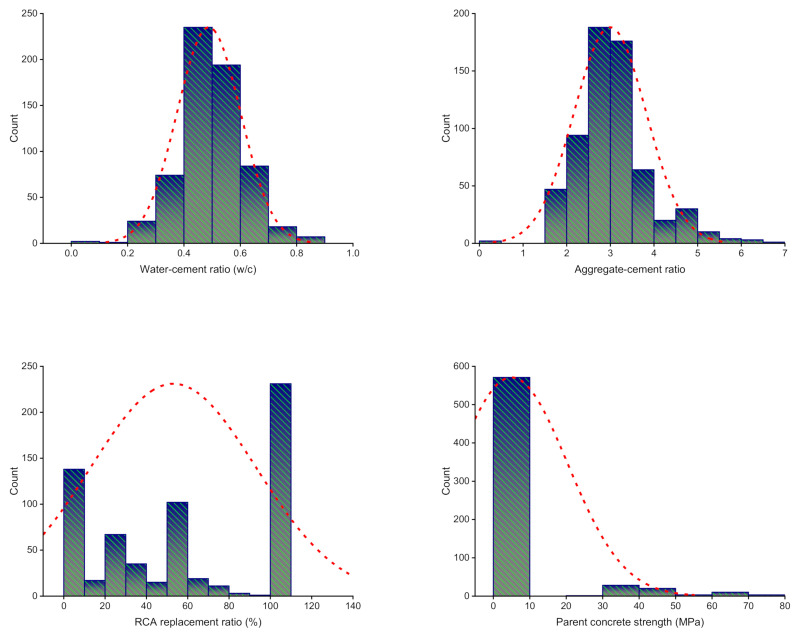

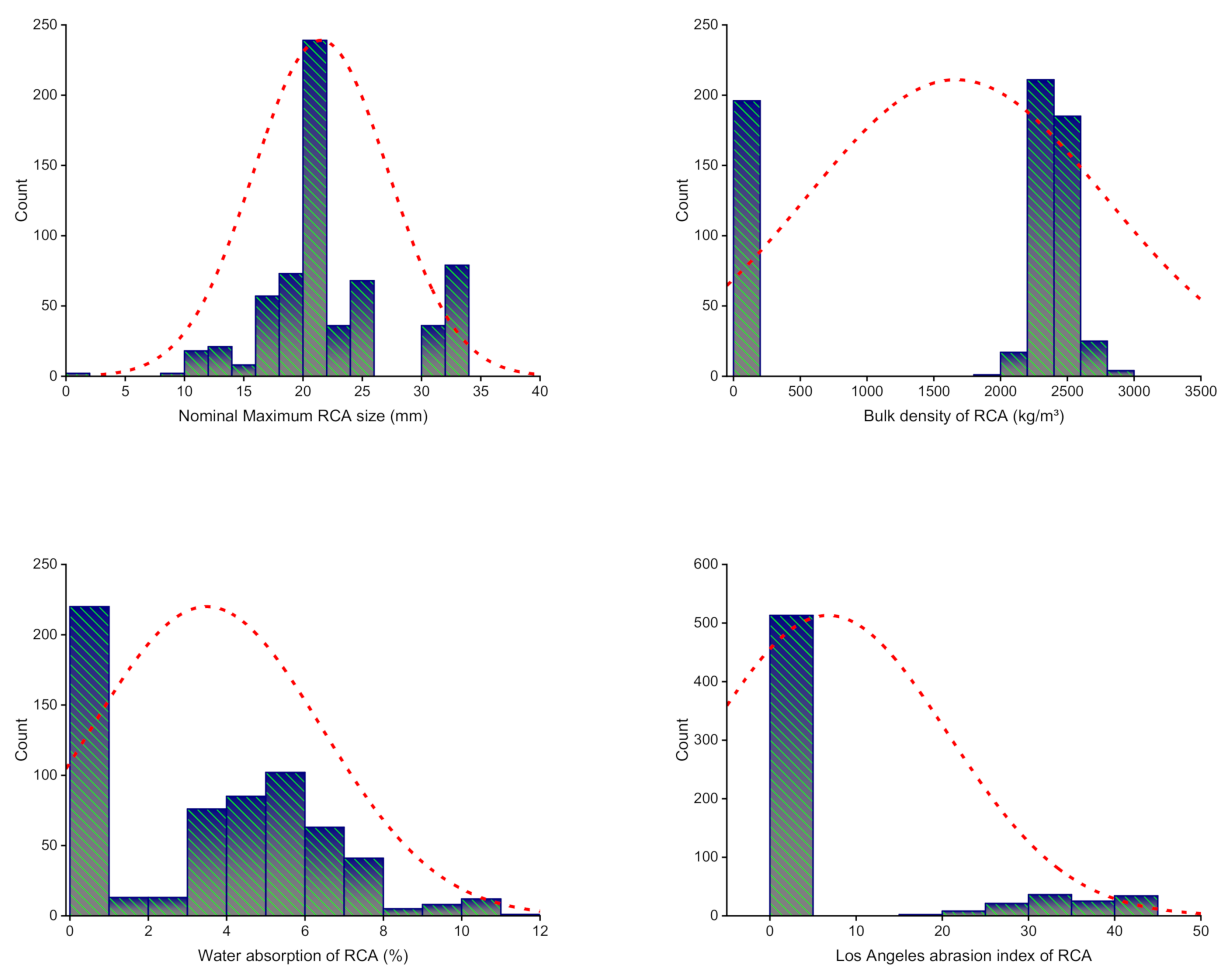
Relative frequency dispersal of input factors.

To attain the desired outcome, ML algorithms require a diverse set of input variables [75,76,77]. Utilizing data gathered from the past studies (see Appendix A), the CS of RAC was calculated. To avoid bias, experimental data were picked at random from past studies. The available publications on the usage of similar materials in the CS of RAC were reviewed. While the majority of articles studied extra aspects of RAC, this analysis used CS data for modeling. The algorithms took eight variables as inputs: the RCA replacement ratio, the parent concrete strength, the aggregate–cement ratio (a/c), the water–cement ratio (w/c), the nominal maximum RCA size, the Los Angeles abrasion index of RCA, the bulk density of RCA, and the water absorption of RCA, and only CS taken as the output. The quantity of input factors and dataset size have a substantial effect on the ML method results [78,79,80]. A total of 638 data points were used in the current research to run ML techniques. The descriptive statistic assessment of all input factors is summarized in Table 1. The table contains the mathematical identifications for all the input factors. Figure 1 depicts the relative frequency dispersal of all variables applied in the investigation. It summarizes the number of possible interpretations for each value or combination of values.

### 2.2. Machine Learning Algorithms Employed

**Figure 2:**
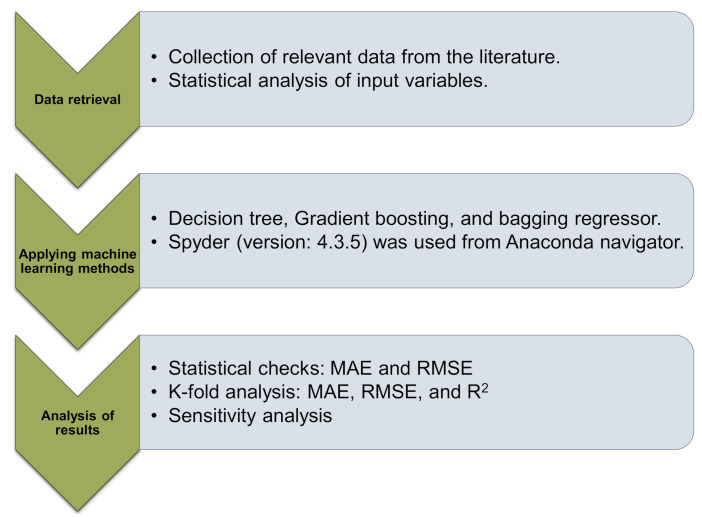
Sequence of research methods.

To meet the study’s aims, a single ML method (DT) and ensemble ML approaches (GB and BR) were employed with Python scripting using the Anaconda Navigator package. Spyder (Version 4.3.5) was selected to operate the DT, GB, and BR models. These ML techniques are frequently utilized to forecast required results in response to input parameters. These techniques are able to anticipate the temperature impact, the mechanical strength, and the durability of materials [81,82,83]. Eight input factors and one output (CS) were used throughout the modeling process. The expected result’s R^2^ score represented the accuracy of all techniques. The R^2^ indicates the degree of deviation; a value near zero indicates greater deviation, while a value near one indicates that the data and model are virtually perfectly fit [70]. The sub-sections beneath describe the ML approaches used in this research. Moreover, statistical and k-fold analyses, as well as error evaluations, are performed on all ML methods like mean absolute error (MAE) and root mean square error (RMSE). Furthermore, sensitivity analysis (SA) is used to determine the influence of all input factors on the estimated results. The research method is depicted in Figure 2.

#### 2.2.1. Decision Tree

**Figure 3:**
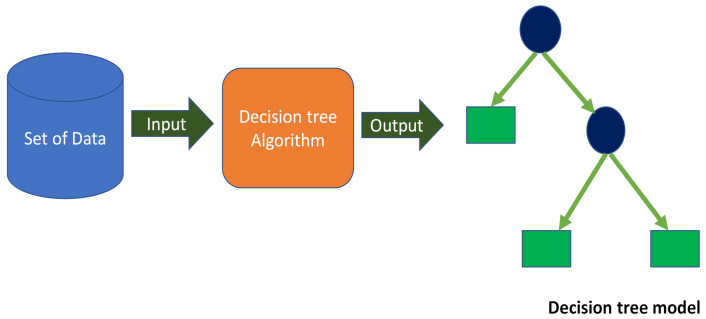
Decision tree schematic representation [85].

DTs are formed by developing techniques for segmenting a data sample into branch-like portions. These portions unite to create an inverted tree with a root node on the upper side [84]. Figure 3 illustrates a schematic representation of the DT technique. As depicted, a DT can have both continuous and single features. Relationships between the object of assessment and the input fields are utilized to generate the branching or segmentation decision rule beneath the root node. Following the link’s establishment, one or more decision rules detailing the associations among the inputs and targeted results might be generated. Decision rules approximate the values of new or undetermined interpretations accurately when they incorporate input values but not targets. At each division point, the errors are computed, and the variable with the smallest fitness function value is taken as the split position, followed by the same for the other variables.

#### 2.2.2. Gradient Boosting

**Figure 4:**
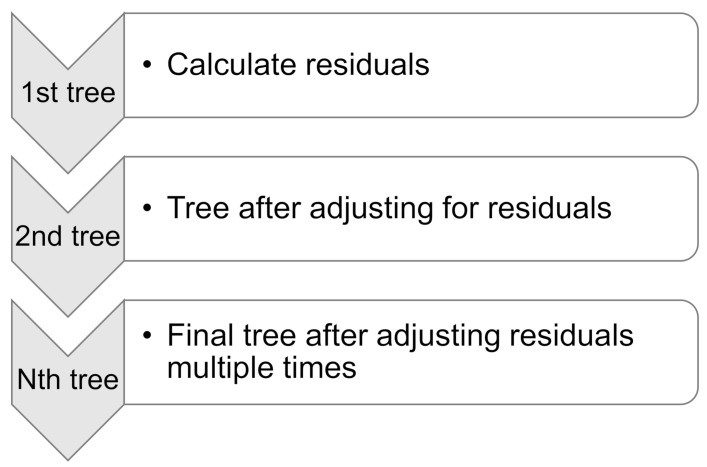
Schematic representation of gradient boosting technique [72].

Friedman [86] presented GB as an ensemble strategy for classification and regression in 1999. GB is only applicable to regression. As seen in Figure 4, the GB technique compares each iteration of the randomly chosen training set to the base model. GB for execution may be sped up and accuracy increased by randomly subsampling the training data, which also helps prevent overfitting. The lower the training data percentage, the faster the regression because the model must suit minor data with every single iteration. The GB algorithm requires tuning parameters, including n-trees and shrinkage rate, where n-trees is the number of trees to be generated; n-trees must not be kept too small, and the shrinkage factor, normally referred to as the learning rate employed to all trees in the development, should not be set too high [87].

#### 2.2.3. Bagging Regressor

**Figure 5:**
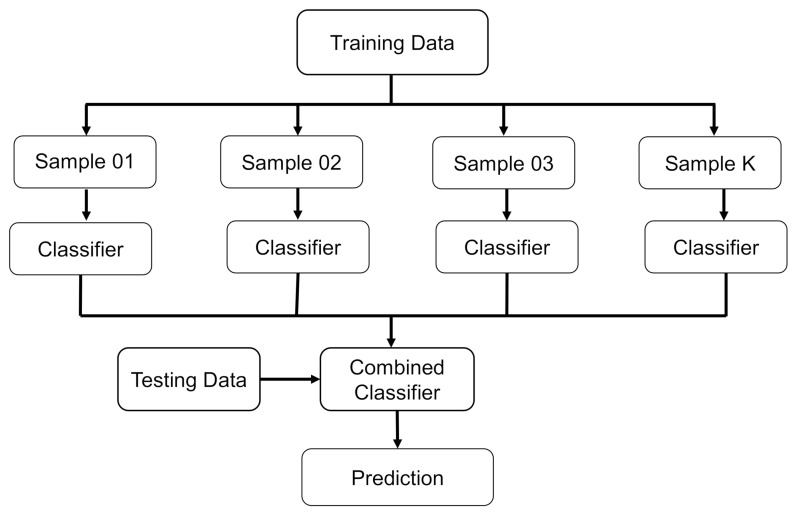
Schematic representation of the bagging regressor technique [85].

BR is a comparable SML technique that compensates for the prediction model’s variance during the training stage by improving it with supplementary data. This result is established on an asymmetric selection strategy that makes use of data exchange from the original set. Utilizing sampling with the substitute, some observations may be reiterated in each new testing dataset, allowing for greater accuracy. During the BR process, each constituent has an equal probability of being included in the new dataset, regardless of its importance. There is no influence on the forecasting force of a training set that is larger in size than the training set. It is also possible to considerably reduce the variation by fine-tuning the estimate to get the desired conclusion. For subsequent model training, each of these data sets is commonly utilized to supplement the others. Using an ensemble of numerous models, the mean of all predictions from each model is used to create this ensemble. In regression, the prediction might be the average or mean of the estimates from a number of different models [88]. Twenty sub-models are employed to optimize the DT using BR to obtain an adamant output result. Figure 5 depicts the bagging algorithm’s flow chart, which details the procedure until the desired output is obtained.

## 3. Analysis of Results

### 3.1. DT Model

**Figure 6:**
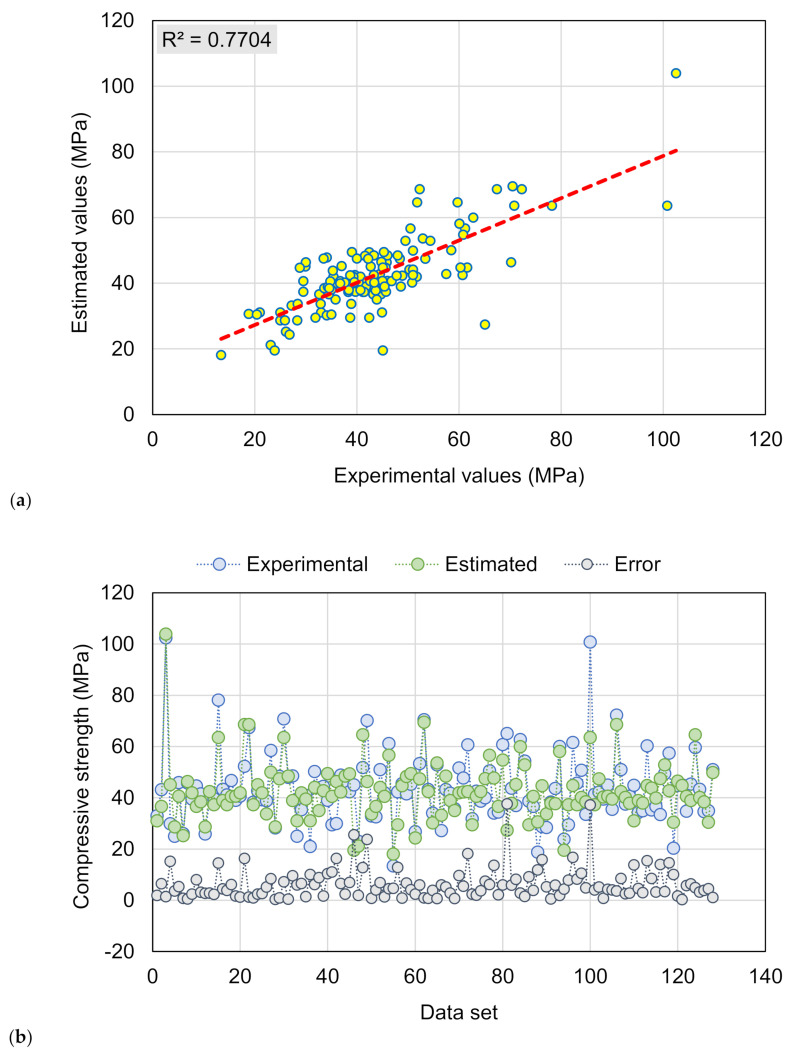
DT model: (**a**) Link among experimental and projected outcomes; (**b**) Scattering of actual and predicted results.

Figure 6 demonstrates the DT model’s results for the CS estimate of RAC. Figure 6a illustrates the relationship among experimental and anticipated results. The DT approach produced findings that were less accurate and had a moderate discrepancy between experimental and projected outcomes. The R^2^ of 0.77 validates the DT model’s lower performance in projecting the CS of RAC. Figure 6b depicts the scattering of experimental, anticipated, and error values for the DT model. The error values were evaluated, and the maximum and average values were noted to be 37.68 and 6.39 MPa, respectively. Furthermore, the dispersal of error values was found, with 11.7% of values falling below 1 MPa, 41.4% falling between 1 and 5 MPa, 28.1% falling between 5 and 10 MPa, and 18.8% falling over 10 MPa. The scattering of error numbers indicates that the DT technique works less precisely.

### 3.2. GB Model

**Figure 7:**
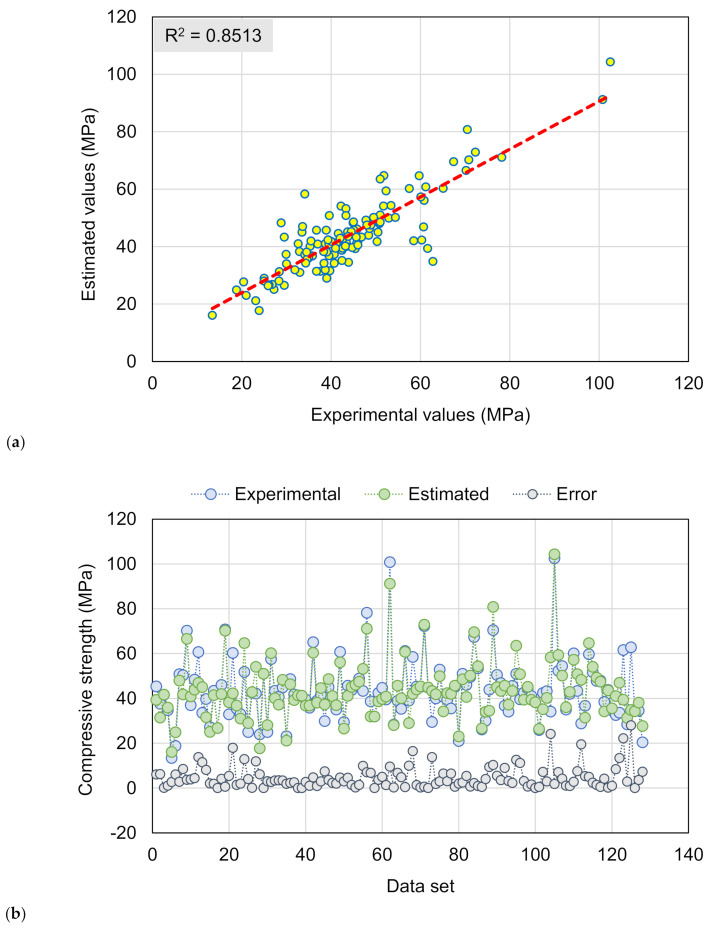
GB model: (**a**) Relationship among experimental and projected outcomes; (**b**) Scattering of actual and predicted results.

Figure 7 shows the outcomes from the GB model’s estimation of the CS of RAC. Figure 7a illustrates the relationship among experimental and estimated results. The GB method resulted in an output that was more precise and had the least degree of difference between actual and projected results. The GB model is better at forecasting the CS of RAC, with an R^2^ of 0.85. The scattering of experimental, anticipated, and error figures for the GB model are depicted in Figure 7b. The results for the average and highest error are 4.78 and 27.96 MPa, respectively. The dispersal of errors was 20.4% lower than 1 MPa, 43.1% in the range of 1 and 5 MPa, 20.0% in the range of 5 and 10 MPa, and 16.5% larger than 10 MPa. The dispersal of errors demonstrates the GB technique’s superior estimating accuracy to the DT. The GB model takes the advantage of optimized value from the twenty sub-models, resulting in the higher precision.

### 3.3. BR Model

**Figure 8:**
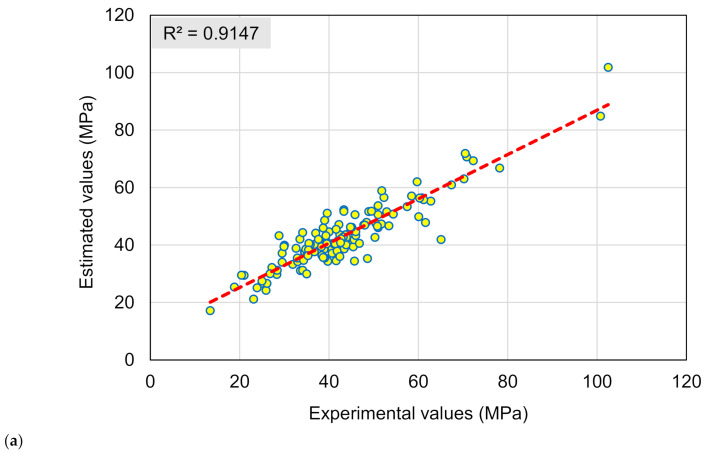

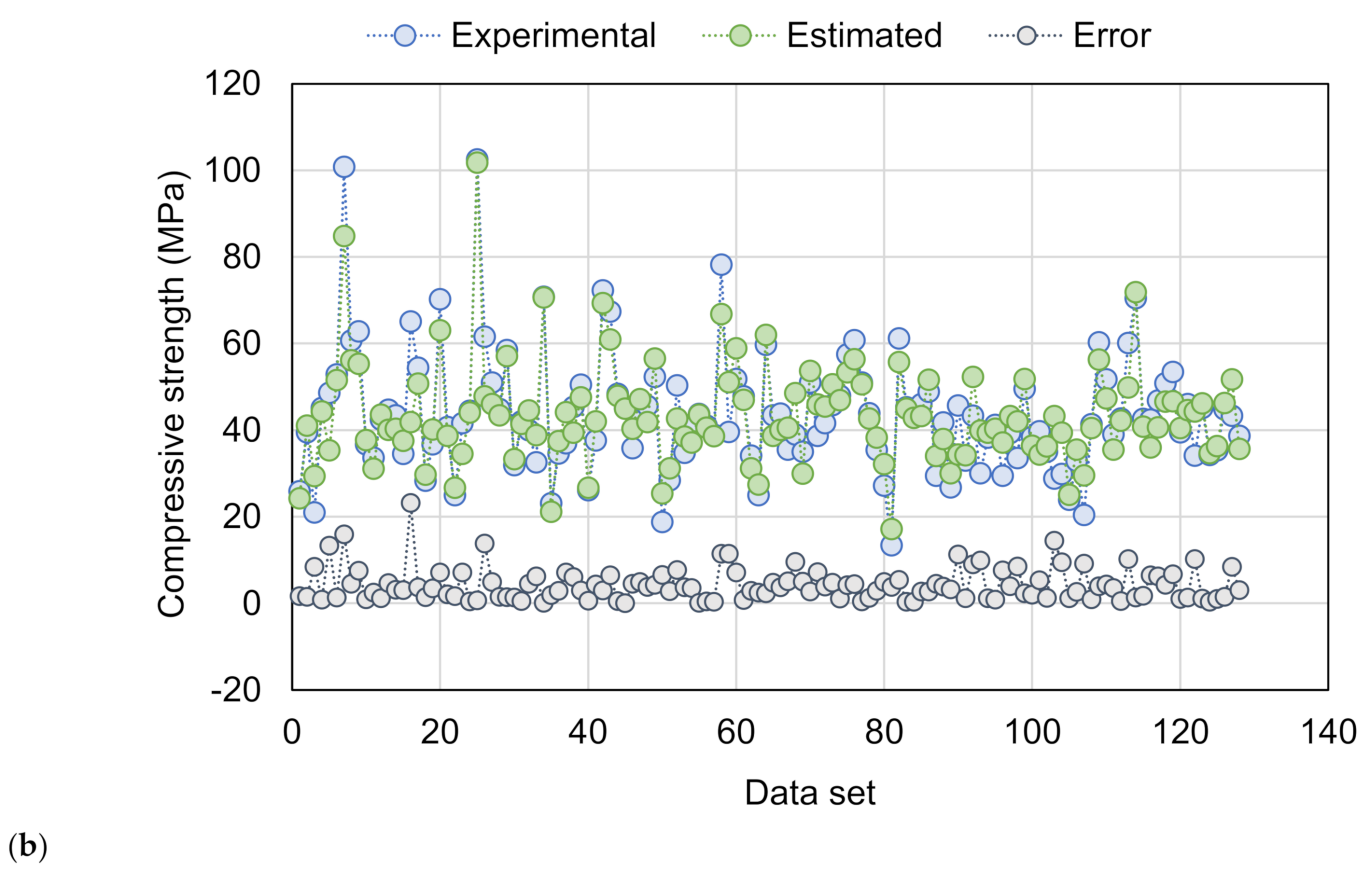
Bagging regressor model: (**a**) Relationship among experimental and forecasted results; (**b**) Scattering of actual and predicted results.

Figure 8a,b exemplify an evaluation of the experimental and expected findings for the BR model. Figure 8a illustrates the relationship among experimental and projected results, with an R^2^ of 0.92 implying that the BR model is more accurate in estimating the RAC’s CS than the DT and GB models. The scattering of experimental, anticipated, and error scores for the BR model are depicted in Figure 8b. The maximum and average errors were found to be 23.22 and 4.26 MPa, respectively. The dispersal of error values was 16.4% lower than 1 MPa, 54.7% in the range of 1 and 5 MPa, 21.15% in the range of 5 and 10 MPa, and only 7.8% higher than 10 MPa. These decreased error numbers suggest that the BR technique is more precise than the other models used in this investigation. Similar to the GB method, the BR method produces twenty sub-models, and the optimized sub-model based on the R^2^ is chosen. Because the BR approach employs substitution sampling, some observations may be repeated in each new testing dataset, resulting in increased accuracy.

## 4. Validation of Models

**Figure 9:**
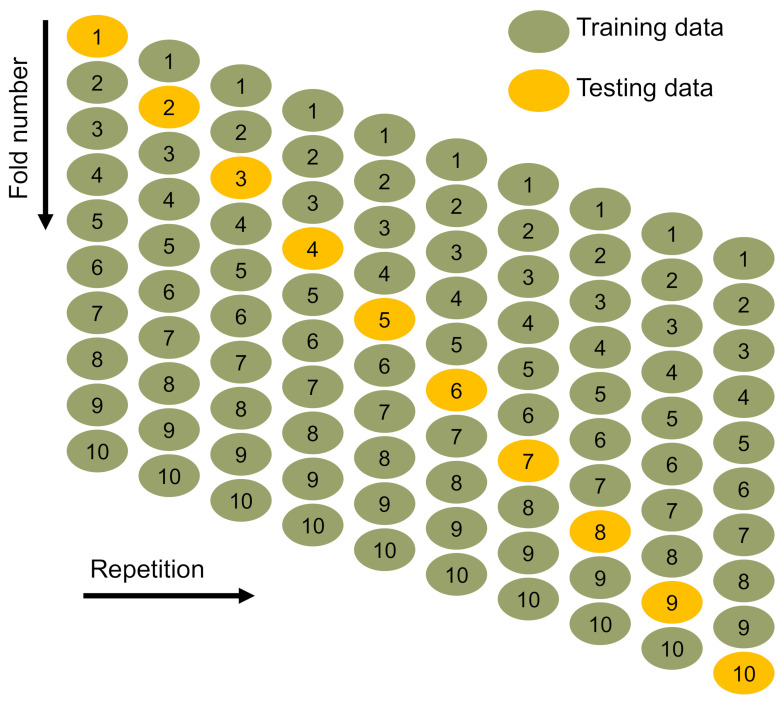
Schematic depiction of k-fold assessment [92].

The models were validated using k-fold and statistical techniques. The k-fold approach is widely used to determine the validity of a technique [89] in which the related dataset is arbitrarily distributed and classified into 10 classes. As depicted in Figure 9, nine units will be utilized for training models and one for verifying them. The model is more accurate when the errors (RMSE and MAE) are small, and the R^2^ is greater. Moreover, the procedure should be repeated ten times to ensure that a plausible conclusion is reached. This substantial effort greatly contributes to the ML technique’s exceptional correctness. Moreover, as seen in Table 2, all ML methods were statistically assessed for the inaccuracy (MAE and RMSE). These analyses also validated the BR model’s superior exactness in comparison to the DT and GB models, owing to their lower error values. The approaches’ predictive performance was assessed statistically using Equations (1) and (2), which were obtained from earlier work [90,91].

(1) $$\mathrm{MAE}\;=\;\frac{1}{n}\overset{n}{\underset{i=1}{\sum }}\left|x_{i}\;-\;x\right|$$

(2) $$\mathrm{RMSE}\;=\;\sqrt{\sum \frac{{\left(y_{pred}\;-\;y_{ref}\right)}^{2}}{n}}$$

where n = total quantity of data points, x, $y_{ref}$ = experimental values in the data set, and $x_{i}$, $y_{pred}$ = projected values from techniques

**Figure 10:**
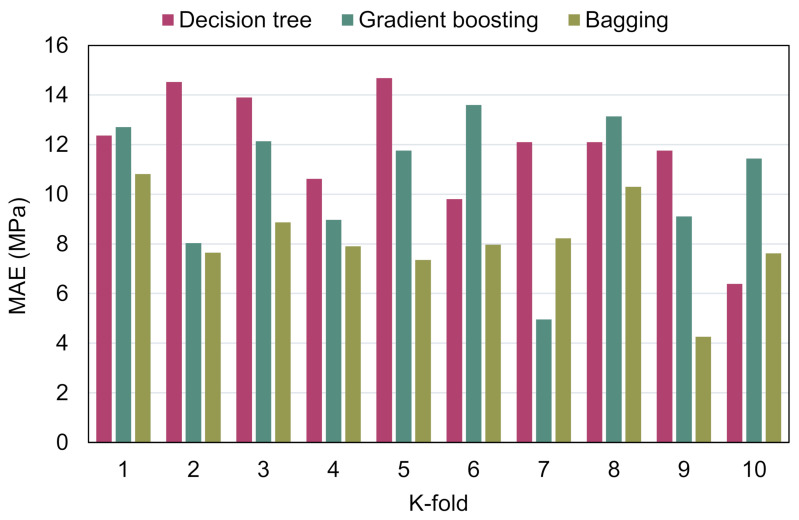
Mean absolute error distribution from k-fold analysis.

**Figure 11:**
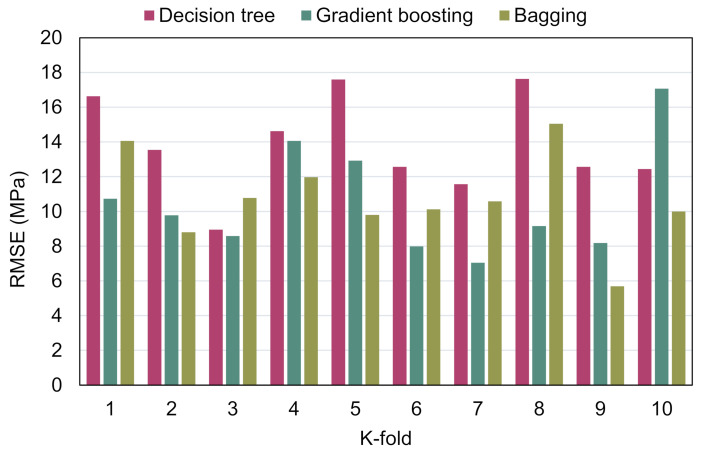
Root mean square error distribution from k-fold analysis.

**Figure 12:**
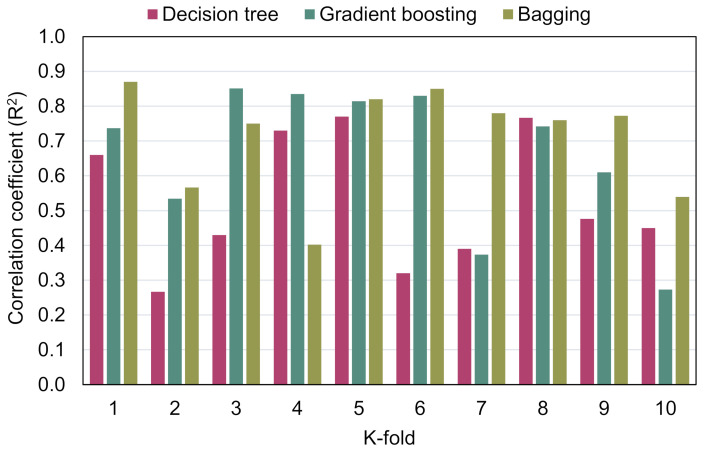
Correlation coefficient (R^2^) distribution from the k-fold analysis.

MAE, RMSE, and R^2^ were computed to determine the effectiveness of the k-fold process, and their values are shown in Table 3. Figure 10, Figure 11 and Figure 12 illustrate the comparison of k-fold analysis for all of the methods used. The MAE for the DT model was in the range of 6.39 and 14.68 MPa, having an average of 11.83 MPa. When compared to the GB method, the MAE varied from 4.78 to 14.60 MPa, having an average of 10.27 MPa. MAE for the BR model ranged from 4.26 to 10.82 MPa, having an average of 8.10 MPa (Figure 10). The average RMSE for the DT, GB, and BR methods were 13.81, 11.05, and 10.69 MPa, respectively (Figure 11). Moreover, the average R^2^ for the DT, GB, and BR models were 0.53, 0.67, and 0.71, respectively (Figure 12). In comparison with the GB and DT methods, the BR method with smaller errors (MAE and RMSE) and superior R^2^ is more exact in estimating the CS of RAC.

## 5. Sensitivity Analysis

**Figure 13:**
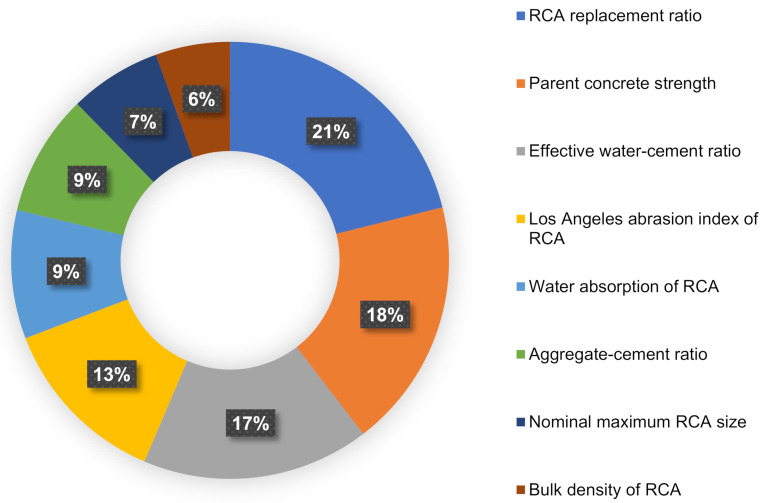
Input variables contribution to predicting outcomes.

This evaluation intends to find out the influence of input factors on RAC’s CS prediction. The input factors have a major influence on the anticipated result [93]. The effect of the input factors on the CS forecast of RAC is seen in Figure 13. The analysis found that the essential ingredient was the RCA replacement ratio, accounting for around 21% of the total, followed by parent concrete strength at approximately 18% and w/c at approximately 17%. The remaining input factors had a smaller effect on the forecast of RAC’s CS, with the Los Angeles abrasion index of RCA, water absorption of RCA, a/c, nominal maximum RCA size, and bulk density of RCA contributing to about 13%, 9%, 9%, 7%, and 6%, respectively. SA produced results that were related to the quantity of inputs and the data sample used to create the models. Equations (3) and (4) were used to determine the effect of an input parameter on the technique’s output.

(3) $$N_{i}\;=\;f_{max}\left(x_{i}\right)\;-\;f_{min}\left(x_{i}\right),$$

(4) $$S_{i}\;=\;\frac{N_{i}}{\sum_{j-i}^{n}N_{j}},$$

where, $f_{max}\left(x_{i}\right)$ is the highest anticipated result over the ith output, $f_{min}\left(x_{i}\right)$ is the least anticipated results over the ith output, and $S_{i}$ is the percentage contribution of a specific input factor.

## 6. Discussion

**Figure 14:**
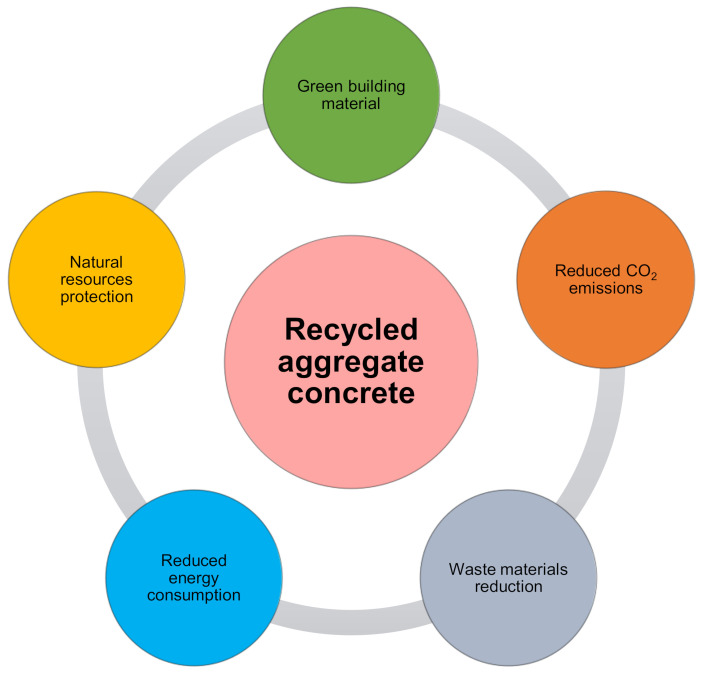
Benefits related to the adoption and application of recycled aggregate concrete.

The goal of this work was to add to the body of knowledge concerning the application of modern strategies for evaluating the CS of RAC. This sort of study will benefit the building sector by facilitating the advancement of fast and cost-efficient material property prediction tools. Furthermore, by encouraging eco-friendly strategies through these measures, the approval and usage of RAC in the building sector will be hastened. Figure 14 illustrates the benefits of RAC in the construction industry. Urbanization and industrialization need considerable infrastructure renewal, resulting in enormous CDW volumes. As a result, landfill area is becoming increasingly scarce as necessary areas are turned into garbage ditches, estate and waste dumping costs continue to rise. As a result, waste management has become a priority in emerging countries and is a worldwide concern that requires a long-term solution. Furthermore, extracting and managing NAs for concrete uses a considerable amount of energy and produces CO_2_ [21]. Thus, including RCA in the manufacturing process of concrete may result in increased energy savings, resource conservation, building sustainability, cost savings, and a large reduction in CDW.

**Figure 15:**
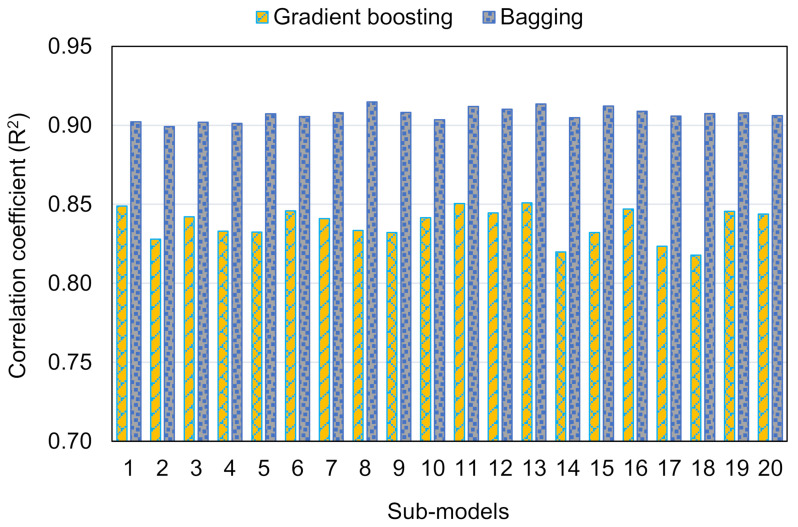
Correlation coefficient of sub-models.

This analysis illustrates how ML strategies might be used to foretell the CS of RAC. Three ML methods, including DT, GB, and BR, were employed. DT is a single ML method, while GB and BR are ensemble ML methods. Each approach was evaluated for exactness to determine the most effective prediction. The BR model, with an R^2^ of 0.92, gave more precise findings than the GB and DT models, which had an R^2^ of 0.85 and 0.77, respectively. Moreover, the accuracy of all techniques was tested by the statistical k-fold analysis techniques. The model’s precision increases as the number of error values decreases. However, defining and suggesting the ideal ML model for forecasting outcomes across several domains is challenging since a model’s precision is highly reliant on the input factors and size of the data set employed during modeling. Ensembled ML methods frequently take advantage of the weak learner by producing sub-models that may be trained on data and tweaked to improve the R^2^. Figure 15 illustrates the dispersion of R^2^ for the GB and BR sub-models. The R^2^ for the GB sub-models were 0.818, 0.844, and 0.869, respectively. Similarly, the R^2^ values for the lowest, average, and maximum BR sub-models were 0.899, 0.907, and 0.915, respectively. These findings indicated that BR sub-models had better R^2^ values than GB sub-models, indicating that the BR model was more precise in estimating RAC’s CS. In addition, an SA was carried out to find out the influence of all input factors on the RAC’s projected CS. The execution of a model might be impacted by the model’s input factors and the quantity of data points. SA was used to find out the contribution of each of the eight input factors to the anticipated output. The three most significant input factors were discovered to be the RCA replacement ratio, parent concrete strength, and w/c.

## 7. Conclusions

The goal of this research was to estimate the compressive strength (CS) of recycled aggregate concrete (RAC) with the application of both single and ensemble machine learning (ML) algorithms. To predict outcomes, a decision tree (DT) and two ensemble approaches—gradient boosting (GB) and bagging regressor BR—were used. As a result of this analysis, the following findings have been drawn:

- Ensemble ML approaches outperformed the single ML approach in estimating the CS of RAC, with the BR model achieving the greatest accuracy. Correlation coefficients (R^2^) were 0.92, 0.85, and 0.77 for the BR, GB, and DT models, respectively. Ensemble ML models (BR and GB) produced findings that were within a reasonable range and did not significantly diverge from experimental results. In comparison, the single ML model (DT) had a lower accuracy and was not suggested for estimating RAC strength.
- The model’s performance was confirmed by statistical tests and k-fold analysis. These evaluations also validated the BR model’s maximum accuracy, as seen by its reduced error values when compared to the GB and DT models.
- Sensitivity analysis indicated that the RCA replacement ratio was the most influential factor determining the model’s outcome, accounting for around 21% of the total, followed by parent concrete strength at around 18% and the water–cement ratio at 16%. However, the other input parameters contributed less to the estimation of RAC’s CS, with Los Angeles abrasion index of RCA, water absorption of RCA, aggregate–cement ratio, nominal maximum RCA size, bulk density of RCA accounting for around 13%, 9%, 9%, 7%, and 6%, respectively.
- This sort of study will benefit the construction industry by allowing for the advancement of rapid and cost-efficient approaches for estimating the strength of materials. Furthermore, by supporting eco-friendly construction through these measures, the adoption and application of RAC in construction will be promoted.

This study suggests that future research should use experimental procedures, mixed proportions, field trials, and other numerical evaluation techniques in order to enhance the number of data points and input parameters. In addition, to improve the models’ responsiveness, environmental factors like temperature and humidity and a complete description of the raw materials may be incorporated as input variables. Furthermore, edge detection methods might be employed to detect cracks in concrete [94,95]. Nevertheless, algorithms for exact product identification and categorization are not confined to edge detection methods. This is a significant restriction of the proposal’s objectives, and its limits should be assumed with greater rigor and realism in developing the arguments for future research.

## Figures and Tables

**Table 1:** Descriptive assessment results of input factors used.

Parameter	Water-Cement Ratio (w/c)	Aggregate-Cement Ratio (a/c)	RCA Replacement Ratio (%)	Parent Concrete Strength (MPa)	Nominal Maximum RCA Size (mm)	Bulk Density of RCA (kg/m^3^)	Water Absorption of RCA (%)	Los Angeles Abrasion Index of RCA
Mean	0.49	2.99	53.03	5.00	21.51	1666.16	3.49	6.75
Maximum	0.87	6.50	100.00	100.00	32.00	2880.00	11.90	42.00
Minimum	0.00	0.00	0.00	0.00	0.00	0.00	0.00	0.00
Range	0.87	6.50	100.00	100.00	32.00	2880.00	11.90	42.00
Mode	0.50	3.10	100.00	0.00	20.00	0.00	0.00	0.00
Median	0.49	2.90	50.00	0.00	20.00	2330.00	3.90	0.00
Sum	312	1913	33,884	3193	13,747	10,646.77	2231	4312
Standard Deviation	0.11	0.83	40.01	15.38	5.71	1115.04	2.94	13.89

**Table 2:** Statistical assessments of the models.

Model	MAE	RMSE
Decision tree	6.389	8.952
Gradient boosting	4.956	7.046
Bagging	4.258	5.693

**Table 3:** Results obtained from the k-fold assessment.

K-Fold	Decision Tree			Gradient Boosting			Bagging Regressor		
	MAE	RMSE	R^2^	MAE	RMSE	R^2^	MAE	RMSE	R^2^
1	12.37	16.63	0.66	12.70	10.73	0.74	10.82	14.06	0.87
2	14.53	13.55	0.27	8.03	9.78	0.53	7.65	8.80	0.57
3	13.90	8.95	0.43	12.14	8.58	0.85	8.87	10.78	0.75
4	10.63	14.62	0.73	8.97	14.06	0.84	7.90	11.97	0.40
5	14.68	17.60	0.77	11.76	12.92	0.81	7.35	9.80	0.82
6	9.80	12.57	0.32	13.60	7.99	0.83	7.97	10.12	0.85
7	12.10	11.57	0.39	4.96	7.05	0.37	8.22	10.58	0.78
8	12.11	17.63	0.77	13.14	9.16	0.74	10.31	15.05	0.76
9	11.76	12.56	0.48	9.11	8.18	0.61	4.26	5.69	0.77
10	6.39	12.44	0.45	11.44	17.06	0.27	7.62	9.99	0.54

## Data Availability

The data used in this research has been properly cited and reported in the main text.

## Appendix A

**Table A1:** Data set retrieved from the literature and used for the analysis.

Refs.	Water-Cement Ratio (w_eff_/c)	Aggregate-Cement Ratio (a/c)	RCA Replacement Ratio (RCA %)	Parent Concrete Strength (MPa)	Nominal Maximum RCA Size (mm)	Bulk Density of RCA (kg/m^3^)	Water Absorption of RCA (%)	Los Angeles Abrasion of RCA	Compressive Strength (MPa)
[96]	0.5	2.6	-	-	20	-	-	-	42.8
	0.5	2.5	20	-	20	-	-	-	42.7
	0.5	2.5	50	-	20	-	-	-	41.3
	0.5	2.3	100	-	20	-	-	-	41.8
[97]	0.45	3.3	-	-	20	-	-	-	51.2
	0.45	3.3	30	-	20	2400	4.9	-	50.6
	0.45	3.3	50	-	20	2400	4.9	-	50.8
	0.45	3.3	100	-	20	2400	4.9	-	50.2
	0.39	2.6	-	-	20	-	-	-	60.3
	0.39	2.6	30	-	20	2400	4.9	-	60.8
	0.39	2.6	50	-	20	2400	4.9	-	61.2
	0.39	2.6	100	-	20	2400	4.9	-	60.2
	0.29	2.2	-	-	20	-	-	-	70.5
	0.29	2.2	30	-	20	2400	4.9	-	70.2
	0.29	2.2	50	-	20	2400	4.9	-	70.8
	0.29	2.2	100	-	20	2400	4.9	-	70
[98]	0.36	2.4	-	41.6	16	-	-	-	48.4
	0.36	2.3	100	41.6	16	-	-	-	44.5
	0.36	2.2	100	41.6	16	-	-	-	38.7
	0.36	2.4	-	50.6	16	-	-	-	48.9
	0.36	2.3	100	50.6	16	-	-	-	46.1
	0.36	2.2	100	50.6	16	-	-	-	42.4
	0.36	2.4	-	63.2	16	-	-	-	48.9
	0.36	2.3	100	63.2	16	-	-	-	52.5
	0.36	2.2	100	63.2	16	-	-	-	50.7
	0.36	2.4	-	35.6	16	-	-	-	48.9
	0.36	2.3	100	35.6	16	-	-	-	45.2
	0.36	2.2	100	35.6	16	-	-	-	42
	0.36	2.4	-	66	16	-	-	-	48.9
	0.36	2.3	100	66	16	-	-	-	49.6
	0.36	2.2	100	66	16	-	-	-	45.1
	0.36	2.7	-	72.3	16	-	-	-	52.3
	0.36	2.4	100	72.3	16	-	-	-	54.4
	0.36	2.3	100	72.3	16	-	-	-	48.2
[99]	0.47	2.5	-	38.4	20	-	-	-	39
	0.47	2.5	15	38.4	20	2410	5.8	-	38.1
	0.45	2.5	30	38.4	20	2410	5.8	-	37
	0.42	2.4	60	38.4	20	2410	5.8	-	35.8
	0.38	2.3	100	38.4	20	2410	5.8	-	34.5
[100]	0.6	4.6	-	-	15	-	-	-	43.5
	0.6	4.1	100	-	15	2450	5.6	-	38.2
	0.45	3.3	-	-	15	-	-	-	61.7
	0.45	2.9	100	-	15	2450	5.6	-	52.8
	0.35	2.6	-	-	15	-	-	-	74.4
	0.35	2.3	100	-	15	2450	5.6	-	62.8
	0.45	3.2	25	-	15	-	-	-	60.7
	0.45	3.1	50	-	15	2450	5.6	-	59.4
[101]	0.57	3.1	-	-	20	-	-	-	48.3
	0.57	3.1	20	-	20	2330	6.3	-	44.9
	0.57	3.1	50	-	20	2330	6.3	-	44.7
	0.57	3	100	-	20	2330	6.3	-	46.8
	0.57	3	-	-	20	-	-	-	40.2
	0.57	3.1	20	-	20	2330	6.3	-	43.2
	0.57	2.9	50	-	20	2330	6.3	-	39.7
	0.57	2.9	100	-	20	2330	6.3	-	43.3
	0.57	3	-	-	20	-	-	-	46
	0.57	2.8	20	-	20	2330	6.3	-	43
	0.57	2.7	50	-	20	2330	6.3	-	38.1
	0.57	2.9	100	-	20	2330	6.3	-	39.1
[102]	0.5	2.4	100	-	25	-	-	-	30.2
	0.5	2.3	100	-	25	-	-	-	36.2
	0.7	3.3	100	-	25	-	-	-	27.7
	0.7	3.2	100	-	25	-	-	-	20.4
[103]	0.43	3	-	-	32	-	-	-	35.9
	0.43	2.9	33	-	32	2520	9.3	-	34.1
	0.43	2.8	53	-	32	2520	9.3	-	29.6
	0.43	2.8	72	-	32	2520	9.3	-	30.3
	0.43	2.7	100	-	32	2520	9.3	-	26.7
[104]	0.42	3	-	-	32	-	-	-	36.8
	0.37	2.9	30	-	32	2442	6	-	37.2
	0.34	2.8	50	-	32	2442	6	-	37.8
	0.38	2	70	-	32	2442	6	-	36.7
	0.27	2.7	100	-	32	2442	6	-	35.2
[105]	0.55	4	-	-	25	-	-	-	42
	0.55	3.9	25	-	25	2430	4.4	-	42
	0.52	3.6	50	-	25	2430	4.4	-	41
	0.5	3.5	100	-	25	2430	4.4	-	40
[106]	0.55	4	-	-	25	-	-	-	35.5
	0.55	3.9	25	-	25	2430	4.5	-	38.8
	0.52	3.6	50	-	25	2430	4.5	-	39.4
	0.5	3.5	100	-	25	2430	4.5	-	38.3
[107]	0.41	3.1	-	-	20	-	-	-	59.4
	0.42	3.2	10	-	20	2165	6.8	-	62.2
	0.43	3.4	20	-	20	2165	6.8	-	58.4
	0.44	3.5	30	-	20	2165	6.8	-	61.3
	0.45	3.7	50	-	20	2165	6.8	-	60.8
	0.45	4.4	100	-	20	2165	6.8	-	61
[108]	0.51	2.6	-	-	20	-	-	-	48.6
	0.49	2.5	20	-	20	2570	3.5	-	45.3
	0.48	2.5	50	-	20	2570	3.5	-	42.5
	0.46	2.5	80	-	20	2570	3.5	-	39.2
	0.45	2.5	100	-	20	2570	3.5	-	37.1
[109]	0.49	4.7	-	-	16	2270	-	-	37.7
	0.49	3.9	100	-	16	2270	-	-	34.6
	0.36	2.4	-	-	16	2270	-	-	57.9
	0.36	2.2	100	-	16	2270	-	-	56.4
	0.49	3.7	-	-	16	2780	-	-	39.8
	0.49	4.4	100	-	16	2780	-	-	40.1
	0.36	2.4	-	-	16	2780	-	-	58.3
	0.36	2.3	100	-	16	2780	-	-	60.2
	0.49	5.1	-	-	16	2565	-	-	40.1
	0.49	4.2	100	-	16	2565	-	-	35.3
	0.36	2.7	-	-	16	2565	-	-	61.8
	0.36	2.4	100	-	16	2565	-	-	57.5
[110]	0.47	3.3	-	-	32	-	-	-	31.2
	0.41	3.3	30	-	32	2449	6	-	31
	0.38	3.2	50	-	32	2449	6	-	29.3
	0.36	3.1	70	-	32	2449	6	-	28.4
	0.32	3	100	-	32	2449	6	-	27.2
[111]	0.45	2.8	-	-	20	-	-	-	66.8
	0.45	2.8	20	-	20	2570	3.5	-	62.4
	0.45	2.7	50	-	20	2570	3.5	-	55.8
	0.45	2.7	100	-	20	2570	3.5	-	42
	0.55	2.6	-	-	20	-	-	-	48.6
	0.55	2.5	20	-	20	2570	3.5	-	45.3
	0.55	2.5	50	-	20	2570	3.5	-	42.5
	0.55	2.5	100	-	20	2570	3.5	-	38.1
[112]	0.65	3.1	-	-	19	-	-	-	21.8
	0.65	3.1	100	-	19	2390	4.4	-	22.1
	0.5	2.9	-	-	19	-	-	-	26.7
	0.5	2.9	100	-	19	2390	4.4	-	25.1
	0.48	2.8	-	-	19	-	-	-	28.9
	0.48	2.8	100	-	19	2390	4.4	-	27.2
	0.43	2.6	-	-	19	-	-	-	31.1
	0.43	2.6	100	-	19	2390	4.4	-	28.7
	0.4	2.4	-	-	19	-	-	-	33.7
	0.4	2.4	100	-	19	2390	4.4	-	29.5
[113]	0.54	3.1	-	-	32	-	-	-	26.8
	0.35	3.1	100	-	32	2512	6.3	-	24.6
	0.49	3.1	100	-	32	2670	1.8	-	26.9
	0.46	2.7	-	-	32	-	-	-	34.3
	0.31	2.7	100	-	32	2512	6.3	-	30.2
	0.43	2.7	100	-	32	2670	1.8	-	34.2
	0.42	2.4	-	-	32	-	-	-	38.6
	0.28	2.4	100	-	32	2512	6.3	-	35.5
	0.39	2.4	100	-	32	2670	1.8	-	38.4
[114]	0.7	4.1	-	-	30	-	-	-	18.1
	0.67	3.9	100	-	30	2520	3.8	34	18
	0.67	3.9	100	-	30	2510	3.9	39	15.4
	0.35	3.1	-	-	30	-	-	-	37.5
	0.35	4.3	100	-	30	2520	3.8	34	36.4
	0.36	2.1	100	-	30	2510	3.9	39	35.7
	0.34	2.1	-	-	30	-	-	-	48.4
	0.34	1.9	100	-	30	2520	3.8	34	44.4
	0.34	2.2	100	-	30	2510	3.9	39	43.8
[115]	0.47	3.3	-	-	32	-	-	-	31.2
	0.41	3.3	30	-	32	2449	6	-	31
	0.38	3.2	50	-	32	2449	6	-	29.3
	0.36	3.1	70	-	32	2449	6	-	28.4
	0.32	3	100	-	32	2449	6	-	27.2
[116]	0.55	2.6	-	-	20	-	-	-	48.6
	0.55	2.6	20	-	20	2580	3.5	-	45.3
	0.55	2.5	50	-	20	2580	3.5	-	42.5
	0.55	2.5	100	-	20	2580	3.5	-	38.1
	0.5	2.6	-	-	20	-	-	-	54.1
	0.5	2.6	20	-	20	2580	3.5	-	51.7
	0.5	2.6	50	-	20	2580	3.5	-	47.1
	0.5	2.6	100	-	20	2580	3.5	-	43.4
	0.45	2.8	-	-	20	-	-	-	66.8
	0.45	2.8	20	-	20	2580	3.5	-	62.4
	0.45	2.7	50	-	20	2580	3.5	-	56.8
	0.45	2.5	100	-	20	2580	3.5	-	52.1
	0.4	2.9	-	-	20	-	-	-	72.3
	0.4	2.8	20	-	20	2580	3.5	-	69.6
	0.4	2.8	50	-	20	2580	3.5	-	65.3
	0.4	2.8	100	-	20	2580	3.5	-	58.5
[117]	0.5	2.9	-	-	25	-	-	-	39.5
	0.5	2.9	30	-	25	2530	1.9	-	36.7
	0.5	2.9	50	-	25	2530	1.9	-	38
	0.5	2.8	100	-	25	2530	1.9	-	36
	0.5	2.8	30	-	25	-	-	-	32.6
	0.5	2.8	50	-	25	2400	6.2	-	30.4
	0.5	2.7	100	-	25	2400	6.2	-	29.5
[118]	0.58	3.2	-	-	32	-	-	-	44.6
	0.52	3.2	50	-	32	2720	4.8	-	41.4
	0.45	3.2	100	-	32	2720	4.8	-	40.7
	0.52	3.2	50	-	32	2650	4.6	-	38.3
	0.46	3.2	100	-	32	2650	4.6	-	36.6
	0.52	3.2	50	-	32	2880	4.4	-	41.2
	0.47	3.2	100	-	32	2880	4.4	-	40.3
[119]	0.41	2.6	-	-	20	-	-	-	42.3
	0.39	2.5	20	-	20	2338	5.2	40.2	47.4
	0.36	2.5	50	-	20	2338	5.2	40.2	47.3
	0.32	2.3	100	-	20	2338	5.2	40.2	54.8
[120]	0.52	2.9	-	-	22	-	-	-	48
	0.52	2.9	10	-	22	-	-	-	46.9
	0.52	2.8	20	-	22	-	-	-	47.7
	0.52	2.8	30	-	22	-	-	-	50.8
	0.52	2.8	40	-	22	-	-	-	48
	0.52	2.8	50	-	22	-	-	-	49.5
	0.52	2.7	100	-	22	-	-	-	50.3
	0.54	3.2	-	-	22	-	-	-	23.5
	0.54	3.1	25	-	22	-	-	-	21.6
	0.54	3.1	100	-	22	-	-	-	20.5
[121]	0.76	4.5	100	-	30	-	-	-	21.1
	0.76	4.5	100	-	30	-	-	-	22
	0.76	4.5	100	-	30	-	-	-	23.1
	0.76	4.5	100	-	30	-	-	-	23.5
	0.76	4.5	100	-	30	-	-	-	20.4
	0.76	4.5	100	-	30	-	-	-	18.9
	0.76	4.5	100	-	30	-	-	-	21.2
	0.66	3.9	100	-	30	-	-	-	25.7
	0.66	3.9	100	-	30	-	-	-	28
	0.66	3.9	100	-	30	-	-	-	25.1
	0.66	3.9	100	-	30	-	-	-	27.5
	0.66	3.9	100	-	30	-	-	-	26.1
	0.66	3.9	100	-	30	-	-	-	27.4
	0.66	3.9	100	-	30	-	-	-	27.7
	0.66	3.9	100	-	30	-	-	-	25
	0.57	3.3	100	-	30	-	-	-	30.5
	0.57	3.3	100	-	30	-	-	-	32.7
	0.57	3.3	100	-	30	-	-	-	32.8
	0.57	3.3	100	-	30	-	-	-	33.1
	0.48	2.7	100	-	30	-	-	-	35.3
	0.48	2.7	100	-	30	-	-	-	35.2
	0.48	2.7	100	-	30	-	-	-	32.5
	0.48	2.7	100	-	30	-	-	-	33.8
	0.41	2.2	100	-	30	-	-	-	41.9
	0.41	2.2	100	-	30	-	-	-	38.4
	0.41	2.2	100	-	30	-	-	-	38.7
	0.41	2.2	100	-	30	-	-	-	41.2
[122]	0.54	3.1	-	-	32	-	-	-	26.8
	0.35	3.1	100	-	32	2512	6.3	-	24.6
	0.49	3.1	100	-	32	2670	1.8	-	26.9
	0.46	3.3	-	-	32	-	-	-	34.3
	0.27	3.3	100	-	32	2512	6.3	-	30.2
	0.41	3.3	100	-	32	2670	1.8	-	34.2
	0.42	3	-	-	32	-	-	-	38.6
	0.24	3	100	-	32	2512	6.3	-	35.5
	0.38	3	100	-	32	2670	1.8	-	38.4
[123]	0.4	3.1	50	-	12	2420	6.8	-	43.3
	0.45	3.1	50	-	12	2400	6.8	-	39.6
	0.5	3.2	50	-	12	2400	6.8	-	38.1
	0.55	3.2	50	-	12	2400	6.8	-	34.5
	0.6	3.3	50	-	12	2400	6.8	-	31.6
	0.4	3.1	50	-	22	2420	8.8	-	46.1
	0.45	3.1	50	-	22	2420	8.8	-	45.8
	0.5	3.2	50	-	22	2420	8.8	-	39.9
	0.55	3.3	50	-	22	2420	8.8	-	36.3
	0.6	3.3	50	-	22	2420	8.8	-	34.7
[124]	0.5	3.5	-	-	20	-	-	-	28.3
	0.5	3.5	20	-	20	2400	-	-	27.2
	0.5	3.5	40	-	20	2400	-	-	26.5
	0.5	3.5	60	-	20	2400	-	-	25.4
	0.5	3.5	80	-	20	2400	-	-	25.1
	0.5	3.5	100	-	20	2400	-	-	20.4
	0.5	3.8	20	-	20	2630	-	-	26.4
	0.5	4.1	40	-	20	2630	-	-	25.9
	0.5	4.5	60	-	20	2630	-	-	23.5
	0.5	4.8	80	-	20	2630	-	-	15.4
[125]	0.51	3.6	-	-	32	-	-	-	43.4
	0.57	3.6	50	-	32	2489	2.4	34	45.2
	0.62	3.6	100	-	32	2489	2.4	34	45.7
[126]	0.65	3.3	-	-	19	-	-	-	20.2
	0.65	3.2	25	-	19	2440	5.8	33.6	18.5
	0.65	3.1	50	-	19	2440	5.8	33.6	18
	0.65	3.1	75	-	19	2440	5.8	-	16.5
	0.42	2.7	-	-	19	-	-	33.6	40
	0.42	2.7	25	-	19	2440	5.8	33.6	33
	0.42	2.6	50	-	19	2440	5.8	33.6	34.5
	0.42	2.5	75	-	19	2440	5.8	33.6	34
[127]	0.65	3.4	-	-	16	-	-	-	31.9
	0.66	3.3	20	-	16	2400	5	-	31.7
	0.68	3.1	50	-	16	2400	5	-	32.4
	0.68	2.8	100	-	16	2400	5	-	30.1
	0.5	2.6	-	-	16	-	-	-	44.8
	0.51	2.5	20	-	16	2400	5	-	43.7
	0.53	2.3	50	-	16	2400	5	-	37.5
	0.56	2.1	100	-	16	2400	5	-	40.5
[128]	0.45	1.9	-	-	19	2420	5.4	-	35.2
	0.45	3.4	64	-	19	2420	5.4	-	41.4
	0.45	2.3	100	-	19	2420	5.4	-	43.9
	0.45	2.1	-	-	19	2500	3.3	-	34.1
	0.45	3.1	64	-	19	2500	3.3	-	44.8
	0.45	2.5	100	-	19	2500	3.3	-	45.9
[129]	0.65	3.4	-	-	16	-	-	-	31.9
	0.65	3.3	20	-	16	2400	5	34	31.7
	0.65	3.1	50	-	16	2400	5	34	32.4
	0.65	2.8	100	-	16	2400	5	34	30.1
	0.5	2.6	-	-	16	-	-	-	44.8
	0.5	2.5	20	-	16	2400	5	34	43.7
	0.5	2.8	50	-	16	2400	5	34	37.5
	0.5	2.1	100	-	16	2400	5	34	40.5
[130]	0.43	3.1	-	-	20	-	-	-	51.8
	0.43	3	25	-	20	2661	1.9	-	47
	0.43	2.9	50	-	20	2602	2.6	-	46
	0.43	2.8	100	-	20	2510	3.9	38.8	42.5
[131]	0.45	2.3	-	-	19	-	-	-	44.4
	0.45	2.3	100	-	19	2490	4.8	37	41
	0.55	2.9	-	-	19	-	-	-	36.7
	0.55	2.9	100	-	19	2490	4.8	37	33.3
	0.65	3.5	-	-	19	-	-	-	30.4
	0.65	3.5	100	-	19	2490	4.8	37	24.8
[132]	0.6	4.6	-	-	19	-	-	-	25
	0.6	4.6	25	-	19	-	-	-	26.7
	0.6	4.5	50	-	19	-	-	-	21.5
	0.6	4.5	75	-	19	-	-	-	21.4
	0.6	4.4	100	-	19	-	-	-	20
	0.45	2.6	-	-	19	-	-	-	39.5
	0.45	2.6	25	-	19	-	-	-	38.3
	0.45	2.5	50	-	19	-	-	-	37
	0.45	2.5	75	-	19	-	-	-	35
	0.45	2.5	100	-	19	-	-	-	33.3
[133]	0.49	3.1	-	-	25	-	-	-	44.3
	0.37	3	100	26.3	25	2490	2.9	-	37.6
	0.43	3	100	42.7	25	2570	2.9	-	43.3
	0.36	2.9	100	42.7	25	2440	5.6	-	42.6
	0.36	2.9	100	65.3	25	2470	5.3	-	44.7
[134]	0.53	6.5	-	-	32	-	-	-	39.3
	0.43	5.4	100	-	32	2263	6	-	33.2
	0.49	5.1	100	-	32	2283	4.2	-	35.6
	0.53	5.1	100	-	32	2292	4.3	-	34.6
	0.6	5.3	100	-	32	2301	5	-	37.3
	0.54	6.4	90	-	32	2609	1.5	-	45.4
	0.46	5.9	60	-	32	2518	2.7	-	54.3
	0.44	5.8	60	-	32	2584	1.6	-	54.4
	0.45	6.4	25	-	32	2594	1.6	-	53.4
[135]	0.43	3	-	-	32	-	-	-	34.8
	0.47	2.9	30	-	32	-	-	-	31.9
	0.49	2.8	50	-	32	-	-	-	30.6
	0.54	2.7	100	-	32	-	-	-	29.7
[136]	0.66	4.6	-	-	20	-	-	-	21
	0.66	4.6	30	-	20	2340	5.3	-	20
	0.61	4.3	50	-	20	2340	5.3	-	19
	0.58	4	100	-	20	2340	5.3	-	18
	0.55	3.8	-	-	20	-	-	-	21
	0.55	3.8	30	-	20	2340	5.3	-	23
	0.51	3.5	50	-	20	2340	5.3	-	24
	0.48	3.4	100	-	20	2340	5.3	-	21
	0.5	3.5	-	-	20	-	-	-	31
	0.5	3.5	30	-	20	2340	5.3	-	25
	0.47	3.2	50	-	20	2340	5.3	-	29
	0.44	3	100	-	20	2340	5.3	-	30
	0.48	3.3	-	-	20	-	-	-	33
	0.48	3.3	30	-	20	2340	5.3	-	39
	0.44	3.1	50	-	20	2340	5.3	-	31
	0.42	2.9	100	-	20	2340	5.3	-	34
[137]	0.6	4.3	-	-	32	-	-	-	36.6
	0.6	3.8	100	-	32	2264	2	-	33.6
	0.52	3.6	-	-	32	-	-	-	41.8
	0.52	3.2	100	-	32	2276	2	-	41.1
	0.47	3	-	-	32	-	-	-	48.6
	0.47	2.7	100	-	32	2273	2	-	48.1
[138]	0.6	3	-	37.3	12	-	-	-	39.5
	0.59	3.2	10	37.3	12	2010	10.9	-	40
	0.57	3.5	30	37.3	12	2010	10.9	-	38.6
	0.54	3.8	50	37.3	12	2010	10.9	-	37.6
	0.46	4.6	100	37.3	12	2010	10.9	-	38.6
	0.45	3.2	-	37.3	12	-	-	-	53.3
	0.44	3.3	10	37.3	12	2010	10.9	-	53.7
	0.42	3.7	30	37.3	12	2010	10.9	-	51
	0.67	4	50	37.3	12	2010	10.9	-	47.8
	0.68	4.8	100	37.3	12	2010	10.9	-	45.1
	0.67	3.3	-	37.3	12	-	-	-	65.2
	0.7	3.4	10	37.3	12	2010	10.9	-	64.6
	0.53	3.8	30	37.3	12	2010	10.9	-	65.4
	0.53	4.1	50	37.3	12	2010	10.9	-	63.2
	0.53	5	100	37.3	12	2010	10.9	-	63
[139]	0.54	3	-	41.4	20	-	-	-	49.8
	0.54	3	20	41.4	20	2451	7.3	40	50.5
	0.54	3	50	41.4	20	2451	7.3	40	48.1
	0.54	2.9	100	41.4	20	2451	7.3	40	45.2
	0.45	3.1	-	41.4	20	-	-	-	59.7
	0.45	3.1	20	41.4	20	2451	7.3	40	64.7
	0.45	3.1	50	41.4	20	2451	7.3	40	55
	0.45	3.1	100	41.4	20	2451	7.3	40	53.9
	0.4	3.2	-	41.4	20	-	-	-	78.7
	0.4	3.2	20	41.4	20	2451	7.3	40	69.9
	0.4	3.2	50	41.4	20	2451	7.3	40	63.8
	0.4	3.1	100	41.4	20	2451	7.3	40	62.8
[140]	0.48	4.1	-	-	10	-	-	-	38.9
	0.48	3.5	100	-	10	2360	4.7	15.1	38.6
	0.39	3.1	100	-	10	2280	6.2	22.1	38.1
	0.29	2.6	100	-	10	2220	7.8	25	39.3
	0.34	2.3	-	-	10	-	-	-	61.9
	0.31	2.1	100	-	10	2360	4.7	15.1	60.1
	0.27	1.8	100	-	10	2280	6.2	22.1	60.2
	0.19	1.5	100	-	10	2220	7.8	25	62.8
[141]	0.52	3	100	-	25	2490	4.9	-	37.6
	0.52	3	100	-	25	2570	2.9	-	43.3
	0.52	2.9	100	-	25	2440	5.6	-	42.6
	0.52	2.9	100	-	25	2470	5.3	-	44.7
	0.52	3.1	-	-	25	-	-	-	44.3
	0.58	3.2	-	-	32	-	-	-	44.6
	0.52	3.2	53	-	32	2720	4.8	-	41.4
[142]	0.58	3.2	100	-	32	2720	4.8	-	40.7
	0.52	3.2	54	-	32	2650	4.6	-	38.3
	0.58	3.2	100	-	32	2650	4.6	-	36.6
	0.52	3.2	53	-	32	2880	4.4	-	41.2
	0.58	3.2	100	-	32	2880	4.4	-	40.3
[143]	0.41	1.7	15	-	20	2330	4.4	-	50.8
	0.41	1.7	30	-	20	2330	4.4	-	44.9
	0.41	1.7	45	-	20	2330	4.4	-	44.6
	0.41	1.7	60	-	20	2330	4.4	-	42.4
	0.41	1.7	15	-	20	2370	4	-	54
	0.41	1.7	30	-	20	2370	4	-	56
	0.41	1.7	45	-	20	2370	4	-	54.4
	0.41	1.7	60	-	20	2370	4	-	40.6
	0.41	1.7	15	-	20	2390	3.6	-	55.2
	0.41	1.7	30	-	20	2390	3.6	-	53.5
	0.41	1.7	45	-	20	2390	3.6	-	56.9
	0.41	1.7	60	-	20	2390	3.6	-	54.7
	0.41	1.7	15	-	20	2320	4.6	-	50.5
	0.41	1.7	30	-	20	2320	4.6	-	48.9
	0.41	1.7	45	-	20	2320	4.6	-	45.8
	0.41	1.7	60	-	20	2320	4.6	-	40
	0.41	1.7	15	-	20	2390	3.7	-	54.4
	0.41	1.7	30	-	20	2390	3.7	-	50.2
	0.41	1.7	45	-	20	2390	3.7	-	49.5
	0.41	1.7	60	-	20	2390	3.7	-	40.4
	0.41	1.7	15	-	20	2390	3.5	-	45
	0.41	1.7	30	-	20	2390	3.5	-	46.9
	0.41	1.7	45	-	20	2390	3.5	-	51.4
	0.41	1.7	60	-	20	2390	3.5	-	53.2
	0.41	1.7	15	-	20	2380	3.8	-	55.3
	0.41	1.7	30	-	20	2380	3.8	-	55.9
	0.41	1.7	45	-	20	2380	3.8	-	52.6
	0.41	1.7	60	-	20	2380	3.8	-	48
	0.41	1.7	15	-	20	2380	3.8	-	49.1
	0.41	1.7	30	-	20	2380	3.8	-	49.9
	0.41	1.7	45	-	20	2380	3.8	-	50.3
	0.41	1.7	60	-	20	2380	3.8	-	47.5
	0.41	1.7	15	-	20	2400	3.5	-	43.2
	0.41	1.7	30	-	20	2400	3.5	-	53.7
	0.41	1.7	45	-	20	2400	3.5	-	50
	0.41	1.7	60	-	20	2400	3.5	-	43.3
	0.41	1.7	15	-	20	2370	4	-	52.9
	0.41	1.7	30	-	20	2370	4	-	49.9
	0.41	1.7	45	-	20	2370	4	-	53.7
	0.41	1.7	60	-	20	2370	4	-	46
[144]	0.48	5.1	-	36	25	-	-	-	41.3
	0.48	5	27	36	25	2250	7	-	51.4
	0.48	4.9	64	36	25	2250	7	-	45.6
	0.48	5	37	36	25	2250	7	-	44.7
	0.48	5	37	36	25	2250	7	-	41.9
[145]	0.5	2.4	100	-	25	2452	4.1	-	51
	0.5	2.4	100	-	25	2452	4.1	-	49
	0.5	2.4	100	-	25	2452	4.1	-	48
	0.5	2.6	-	-	25	2452	4.1	-	52
	0.5	2.5	50	-	25	2452	4.1	-	51
	0.5	2.5	50	-	25	2452	4.1	-	51
	0.5	2.5	50	-	25	2452	4.1	-	51
	0.5	2.5	25	-	25	2452	4.1	-	52
	0.5	2.5	25	-	25	2452	4.1	-	50
	0.5	2.5	25	-	25	2452	4.1	-	49
[146]	0.38	2	-	-	25	-	-	-	54.1
	0.28	2	100	-	25	2260	7.5	-	38.3
	0.28	2	100	-	25	2260	7.5	-	32.9
	0.23	2	100	-	25	2260	7.5	-	33.2
	0.46	2.4	-	-	25	-	-	-	42.2
	0.34	2.4	100	-	25	2260	7.5	-	31.3
	0.34	2.4	100	-	25	2260	7.5	-	28.4
	0.28	2.4	100	-	25	2260	7.5	-	28
	0.58	3.1	-	-	25	-	-	-	28.8
	0.43	3.1	100	-	25	2260	7.5	-	26.5
	0.43	3.1	100	-	25	2260	7.5	-	23.3
	0.35	3.1	100	-	25	2260	7.5	-	21.6
	0.67	3.5	-	-	25	-	-	-	23.6
	0.49	3.5	100	-	25	2260	7.5	-	21.6
	0.49	3.5	100	-	25	2260	7.5	-	18
	0.4	3.5	100	-	25	2260	7.5	-	18.8
	0.8	4.2	-	-	25	-	-	-	17.3
	0.59	4.2	100	-	25	2260	7.5	-	16.1
	0.59	4.2	100	-	25	2260	7.5	-	13.4
	0.48	4.2	100	-	25	2260	7.5	-	13.9
[147]	0.6	3.6	-	-	20	-	-	-	38
	-	-	-	-	-	-	-	-	-
	0.59	3.3	20	-	20	2320	5.3	42	41
	0.57	3.3	50	-	20	2320	5.3	42	44
	0.54	3	100	-	20	2320	5.3	42	45
	0.46	2.6	-	-	20	-	-	42	51.5
	0.45	2.5	20	-	20	2320	5.3	-	50.5
	0.44	2.5	50	-	20	2320	5.3	42	45
	0.42	2.3	100	-	20	2320	5.3	42	56
	0.67	3.6	-	-	20	-	-	-	37
	0.68	3.4	20	-	20	2320	5.3	42	33.5
	0.67	3	50	-	20	2320	5.3	42	32
	0.7	2.3	100	-	20	2320	5.3	42	32
	0.53	2.7	-	-	20	-	-	-	45
	0.53	2.5	20	-	20	2320	5.3	42	44
	0.53	2.2	50	-	20	2320	5.3	42	41
	0.52	1.8	100	-	20	2320	5.3	42	41.5
	0.51	3.1	-	-	20	-	-	-	46.5
	0.52	3.2	20	-	20	2320	5.3	42	44
	0.54	3	50	-	20	2320	5.3	42	41
	0.58	2.8	100	-	20	2320	5.3	42	33.5
	0.42	2.7	-	-	20	-	-	-	58
	0.42	2.9	20	-	20	2320	5.3	42	53.5
	0.44	2.7	50	-	20	2320	5.3	42	54
	0.49	2.5	100	-	20	2320	5.3	42	40
[148]	0.42	2.6	50	-	20	2330	6.1	34.6	41.6
	0.51	2.3	100	-	20	2330	6.1	34.6	31.4
	0.52	2.6	50	-	20	2330	6.1	34.6	35.5
	0.61	2.3	100	-	20	2330	6.1	34.6	26
	0.44	2.6	50	-	20	2320	5.8	32.2	44.6
	0.51	2.3	100	-	20	2320	5.8	32.2	36.7
	0.62	2.3	100	-	20	2320	5.8	32.2	29.5
	0.41	2.8	20	-	20	2360	3.9	30.8	46.1
	0.42	2.6	50	-	20	2360	3.9	30.8	45.1
	0.45	2.3	100	-	20	2360	3.9	30.8	42.9
	0.5	2.8	20	-	20	2360	3.9	30.8	39.3
	0.52	2.6	50	-	20	2360	3.9	30.8	39.5
	0.54	2.3	100	-	20	2360	3.9	30.8	37.7
	0.42	2.8	20	-	20	2350	4.5	28.5	48.1
	0.43	2.6	50	-	20	2350	4.5	28.5	41
	0.4	2.3	100	-	20	2350	4.5	28.5	38.7
	0.51	2.8	20	-	20	2350	4.5	28.5	42.7
	0.52	2.6	50	-	20	2350	4.5	28.5	35.4
	0.5	2.3	100	-	20	2350	4.5	28.5	31.4
	0.42	2.8	20	-	20	2350	4.7	30.1	48.5
	0.42	2.6	50	-	20	2350	4.7	30.1	45.4
	0.43	2.3	100	-	20	2350	4.7	30.1	37
	0.52	2.8	20	-	20	2350	4.7	30.1	41.3
	0.52	2.6	50	-	20	2350	4.7	30.1	36.8
	0.56	2.3	100	-	20	2350	4.7	30.1	31.2
[149]	0.41	2.6	-	-	32	-	-	-	47.2
	0.38	2.6	33	-	32	2578	9.3	-	42.4
	0.36	2.6	53	-	32	2578	9.3	-	45.7
	0.34	2.6	72	-	32	2578	9.3	-	36.7
	0.31	2.6	100	-	32	2578	9.3	-	38.9
[150]	0.47	3.8	-	-	20	-	-	-	53.1
	0.47	3.7	20	-	20	2336	3.6	-	50
	0.47	3.6	50	-	20	2315	3.6	-	45.3
	0.47	3.6	75	-	20	2295	3.6	-	44
	0.47	3.5	100	-	20	2273	3.6	-	41.6
[151]	0.29	2.9	-	-	10	-	-	-	102.1
	0.29	2.8	20	100	10	2470	3.7	24	108
	0.29	2.8	50	100	10	2470	3.7	24	104.8
	0.29	2.7	100	100	10	2470	3.7	24	108.5
	0.29	2.8	20	60	10	2390	4.9	25.2	102.5
	0.29	2.7	50	60	10	2390	4.9	25.2	103.1
	0.29	2.6	100	60	10	2390	4.9	25.2	100.8
	0.29	2.8	20	40	10	2300	5.9	24.3	104.3
	0.29	2.7	50	40	10	2300	5.9	24.3	96.8
	0.29	2.5	100	40	10	2300	5.9	24.3	91.2
[152]	0.65	4.6	-	-	20	-	-	-	18
	0.65	4.7	25	-	20	2380	6.94	29	14.7
	0.65	4.8	50	-	20	2380	6.94	29	14.6
	0.65	4.8	75	-	20	2380	6.94	29	14.2
	0.72	5.8	-	-	20	-	-	-	30.8
	0.72	5.9	20	-	20	2380	6.94	29	26.8
	0.72	6	40	-	20	2380	6.94	29	26.6
	0.45	1.9	-	-	16	-	-	-	66.9
	0.45	2.3	20	-	16	2380	6.94	29	49.3
	0.45	2.5	40	-	16	2380	6.94	29	40.9
[153]	0.6	3.5	-	-	16	-	-	-	42
	0.6	3.4	20	-	16	2380	6.9	-	42.9
	0.6	3.4	50	-	16	2380	6.9	-	42.5
	0.6	3.2	100	-	16	2380	6.9	-	40.9
	0.5	2.7	-	-	16	-	-	-	50.2
	0.5	2.6	20	-	16	2380	6.9	-	51.6
	0.5	2.5	50	-	16	2380	6.9	-	51.6
	0.5	2.4	100	-	16	2380	6.9	-	50.3
[154]	0.5	3.4	-	-	22	-	-	-	46.7
	0.5	3.4	50	-	12	2380	-	-	46.9
	0.5	3.4	50	-	22	2380	-	-	46.4
	0.5	3.4	100	-	22	2380	-	-	48.6
[155]	0.52	2.2	-	-	19	-	-	-	29.9
	0.49	2.1	25	-	19	2500	6.6	-	32.6
[156]	0.5	3.5	50	-	8	2330	3.8	41.4	33
	-	-	-	-	-	-	-	-	-
[157]	0.5	3.2	50	-	8	2280	4.1	-	29.1
	0.68	3.8	-	-	20	-	-	-	34.5
	0.68	3.6	100	-	20	2450	3.1	-	35
	0.68	3.4	100	-	20	2370	7.1	-	29.2
	0.68	3.4	100	-	20	2360	7.8	-	27.7
	0.51	3.3	-	-	20	-	-	-	48.3
	0.51	3.1	100	-	20	2450	3.1	-	47.6
	0.51	3	100	-	20	2370	7.1	-	42
	0.51	3	100	-	20	2360	7.8	-	42.9
	0.44	2.5	-	-	20	-	-	-	61.6
	0.44	2.4	100	-	20	2450	3.1	-	60
	0.44	2.3	100	-	20	2370	7.1	-	53.7
	0.44	2.3	100	-	20	2360	7.8	-	53.2
	0.34	2.2	-	-	20	-	-	-	80.8
	0.34	2.1	100	-	20	2450	3.1	-	78.2
	0.34	2	100	-	20	2370	7.1	-	71.2
[158]	0.34	2	100	-	20	2360	7.8	-	65.4
	0.5	3.1	-	-	19	-	-	-	36.5
	0.5	3	30	-	19	2570	2.7	-	33.6
	0.5	3	60	-	19	2570	2.7	-	30.4
[159]	0.5	2.8	100	-	19	2570	2.7	-	29.1
	0.65	3.1	-	-	20	-	-	-	40.5
	0.65	3.2	20	-	20	2300	5.2	40.2	39.5
	0.65	3.2	50	-	20	2300	5.2	40.2	40.8
	0.65	3.2	100	-	20	2300	5.2	40.2	43.7
	0.65	3.1	-	-	20	-	-	-	40.5
	0.65	3.1	20	-	20	2300	5.5	28.6	41
	0.65	3.1	50	-	20	2300	5.5	28.6	38.8
[160]	0.65	3.2	100	-	20	2300	5.5	28.6	39.9
	0.42	2.7	-	-	25	-	-	-	38.6
	0.4	2.7	16	-	25	2200	5.4	-	32.7
	0.39	2.2	37	-	25	2200	5.4	-	31.7
[161]	0.36	2.7	52	-	25	2200	5.4	-	29
	0.86	4.6	-	-	22	-	-	-	23.9
	0.65	3.4	-	-	22	-	-	-	38.7
	0.41	2.9	-	-	22	-	-	-	71.1
	0.87	4.6	100	-	22	2451	7.8	-	19.7
	0.66	3.4	100	-	22	2387	6.9	-	35.7
	0.42	2.8	100	-	22	2362	4.2	-	66.8
	0.86	4.6	100	-	22	2456	7.5	-	21.8
	0.65	3.5	100	-	22	2455	6.4	-	36.1
	0.42	2.9	100	-	22	2496	4.2	-	68.5
	0.81	4.9	-	-	22	-	-	-	27.5
	0.63	3.6	-	-	22	-	-	-	42.4
	0.4	3	-	-	22	-	-	-	72.3
	0.84	4.5	100	-	22	2401	7.6	-	21
	0.63	3.5	100	-	22	2484	5.4	-	41.1
	0.4	2.8	100	-	22	2363	3.6	-	70.2
	0.82	4.7	100	-	22	2447	6.9	-	23.6
	0.64	3.4	100	-	22	2458	5.8	-	39.7
[162]	0.42	2.9	100	-	22	2464	3.9	-	66.5
	0.64	3	-	-	19	-	-	-	33
	0.77	3.1	100	-	19	2268	4.9	-	27.5
[163]	0.7	3.4	100	-	19	1946	11.9	-	29.9
	0.6	3.6	-	-	19	-	-	-	47.8
	0.59	3.3	20	-	19	2320	5.3	37	49.3
	0.57	3.3	50	-	19	2320	5.3	37	47.5
	0.54	3	100	-	19	2320	5.3	37	53.7
	0.46	2.6	-	-	19	-	-	-	62
	0.45	2.5	20	-	19	2320	5.3	37	64.8
	0.44	2.5	50	-	19	2320	5.3	37	63.5
	0.42	2.3	100	-	19	2320	5.3	37	65.1
	0.67	3.6	-	-	19	-	-	-	62
	0.68	3.4	20	-	19	2320	5.3	37	64.8
	0.67	3	50	-	19	2320	5.3	37	63.5
	0.7	2.3	100	-	19	2320	5.3	37	65.1
	0.53	2.7	-	-	19	-	-	-	57.3
	0.53	2.5	20	-	19	2320	5.3	37	54.9
	0.53	2.2	50	-	19	2320	5.3	37	51.5
	0.52	1.8	100	-	19	2320	5.3	37	50.3
	0.51	3.1	-	-	19	-	-	-	60.1
	0.52	3.2	20	-	19	2320	5.3	37	56.5
	0.54	3	50	-	19	2320	5.3	37	48.9
	0.58	2.8	100	-	19	2320	5.3	37	43.1
	0.42	2.7	-	-	19	-	-	-	72.9
	0.42	2.9	20	-	19	2320	5.3	37	67.4
	0.44	2.7	50	-	19	2320	5.3	37	61.2
